# Closed reduction and intramedullary pinning in the treatment of adult radial neck fractures: a case report

**DOI:** 10.11604/pamj.2015.20.434.6580

**Published:** 2015-04-30

**Authors:** Sancar Serbest, Murat Gürger, Haci Bayram Tosun, Lokman Karakurt

**Affiliations:** 1 Department of Orthopaedics and Traumatology, Faculty of Medicine, Kirikkale University, Kirikkale, Turkey; 2 Department of Orthopaedics and Traumatology, Faculty of Medicine, Firat University, Elazig, Turkey; 3 Department of Orthopaedics and Traumatology, Faculty of Medicine, Adiyaman University, Adiyaman, Turkey

**Keywords:** Radial neck, fractures, closed reduction, intramedullary pinning, treatment

## Abstract

Closed reduction and intramedullary pinning (CIMP) in pediatric radial neck fractureswas first reported by Metaizeau in 1980 andsatisfactory results have been published several times. The current literature did not encounter any publication related to the implementation of Metaizeau method to adult patients. We applied Metaizeau technique to an adult radial neck fracture and we have achieved satisfactory results. As this case report is single case of this method applied to an adult, we decided to present this case.

## Introduction

Radial neck fractures often occur as a result of falls onto an outstretched hand with elbow in extension [1]. After falling, radial head is compressed by humeral condyle with the effect of the physiological cubitus valgus while the load is transferred to the elbow through radial body. As a result of this, fracture develops in the more fragile subcapital area. This lesion may be accompanied by cartilage lesions in radial head or humeral condyles, lateral ligament injury, elbow dislocation and inferior radio-cubital separation [2]. Displaced and angled radial neck fractures are usually treated with open reduction and internal fixation method in adults [3].

## Patient and observation

**Figure 1:**
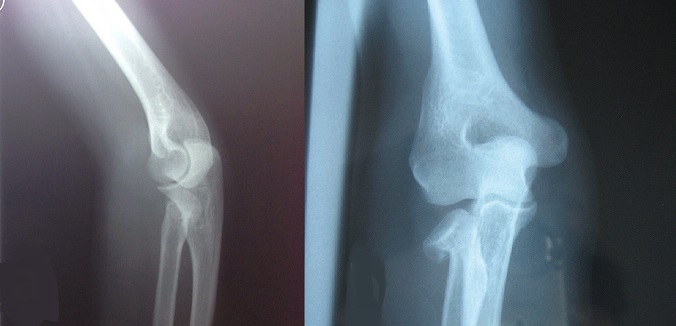
Preoperative AP and lateral view of the elbow

**Figure 2:**
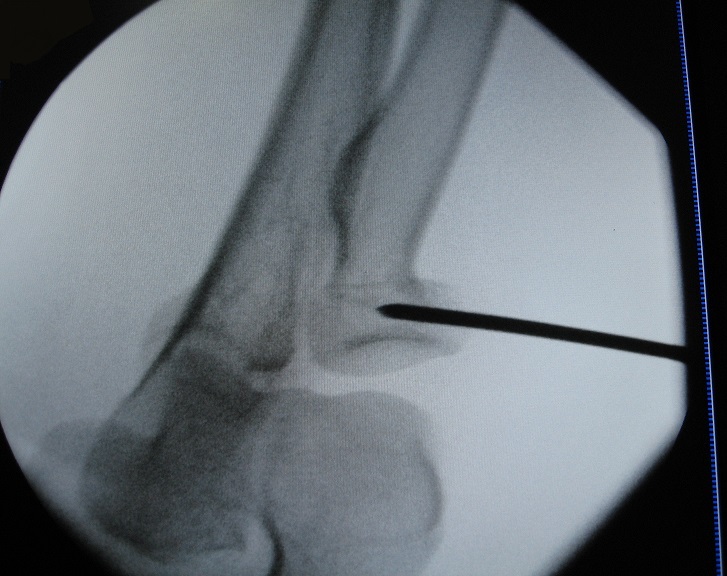
Adequate reduction by percutaneous external K-wire

A 37-year-old male patient admitted to the emergency department with swelling of elbow, limitation of movement and pain after falling onto palm with elbow in extension. Patient's radiographs revealed displaced and angled radial neck fracture (Figure 1). The angle between radial neck and long axis was measured as 52 degrees. MRI was performed in order to evaluate possible accompanying ligament and soft tissue damage at elbow and its surrounding and chondral damage. No bony, soft tissue and chondral damage were detected on MRI. Only radial neck fracture was present in the patient. There upon surgery was planned. Closed reduction under general anesthesia was planned first. Reduction could not be achieved with twice closed reduction attempts; so closed reduction with intramedullary pinning was decided. One K-wire, which can be used as a joystick, was inserted into radial head under fluoroscopic controlin the transverse plane to achieve reduction indirectly (Figure 2). Then cortex was reached following 1 cm skin incision over lateral radial distal metaphysis with great care taken not to injure the sensory branch of the radial nerve; a 3.2 drill is introduced to cortex to open a window. A T-handle was placed to approximately 30^°^angled, 1 cm distal end of a 2-millimeters thick, 25 cm long K-wire. K-wire was introduced over the entrance point under fluoroscopic control with concavity facing outside and elbow in extension and traction.

**Figure 3:**
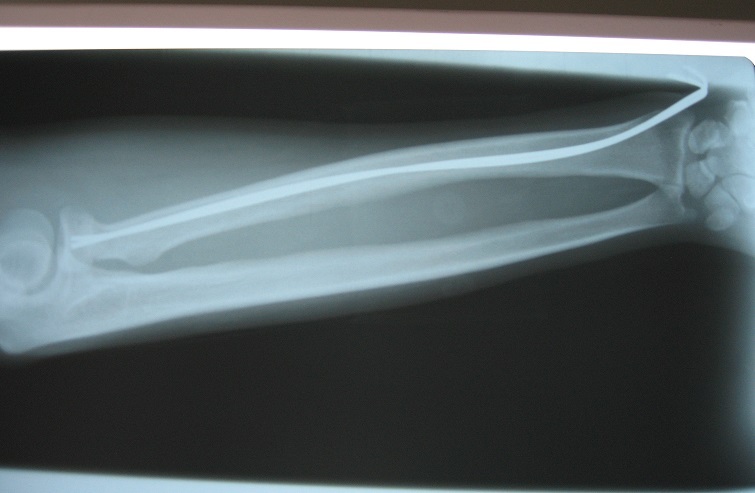
Intramedullary K-wire is pushed upwards and has been twisted so that it points face inward, the lateral shift is corrected

**Figure 4:**
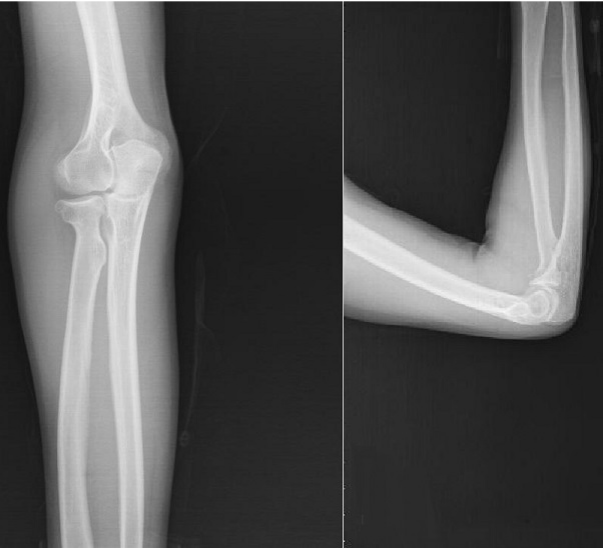
AP and lateral projections of elbow 1 year after the fracture

**Figure 5:**
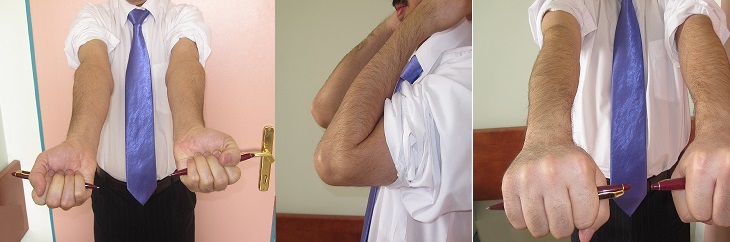
Full functional movement of the elbow with excellent result

K-wire was advanced into the proximal metaphyseal-diaphyseal region. Meantime joystick K-wire that was inserted to radial head in the transverse plane was removed. K-wire was advanced upwards, attached to metaphyseal residues of radial head and the wire was rotated 180^°^ untilreduction was achieved. Mean while, the radial head was checked not to be drilled and cause chondral damage. Thus radial head at the end of the wire was allowed to return to the interior by being dragged. K-wire was stopped by lateral humeral condyle, which serves as a tampon, while advancing toward radial head. In this way, excessive correction was prevented. As the patient was adult, a second intramedullary 1.8-mm K-wire was inserted to achieve a more stable fixation (Figure 3). Above elbow circular cast was applied for two weeks to complete elbow recovery. Then active movements were started. No strenuous exercise was given to patients. Radiological control was performed at intervals of three weeks. Bone healing could be visualized radiographically at third month. Intramedullary pin was removed from the patient under local anesthesia in the outpatient clinic after 4 weeks (Figure 4). Range of motion of the elbowjoint was 145 degrees for flexion, 90 degrees for supination and 90 degrees for pronation. Elbow joint was pain-free (Figure 5).

## Discussion

Radial head and neck fractures are the most common fractures of the elbow. They constitute 2-5% of all fractures [4]. Radial head and neck fractures constitute 33-25% of elbow fractures [3]. The treatment and outcome of radial head and neck fractures depend on the size of the fracture fragments, the integrity of the articular surface, intra-articular fragment and the angle between radial neck and radial shaft [5]. Pediatric radial neck fractures with less than 30 degrees angulation and without displacement can be treated conservatively. In this case, there is no need for any manipulation. If fracture is angulated more than 30 degrees, manipulation under general anesthesiato achieve a better alignment is recommended. In case of over sixty degrees or excessive displacement, surgery must be performed to ensure adequate reduction [6]. Again in children, reduction of fracture is also possible by pushing the fragment with a percutaneously inserted K-wire under fluoroscopic control. In cases where closed reduction and percutaneous pinning failed, open reduction is recommended [2]. Alsovery good results have been achieved with closed reduction and intramedullary pinning (CIMP) technique reported by Metaizeau in these fractures in children. CIMP method is fast, easy, and has a short learning period. Advantages are that manipulation is extra-articular and surgery is minimally invasive [7].

In the treatment of adults, surgery can be decided aftera careful evaluation of x-rays andradio-humeral joint aspiration and the examination of the range of motion of pronation and supination after local anesthetic injection. If 70 degrees of active pronation and supination movements can be achieved, conservative treatment can be tried without taking X-rays into consideration. Radial head excisionis also a treatment option for patients other than children. Isolated large non-comminuted radial head and neck fractures can be treated with open reduction internal fixation using mini AO screws, Herbert screws or Acutrac screws [8]. Complications are nonunion, avascular necrosis, radioulnar synostosis and the development of myositis ossificans; delay in treatmentand open reduction internal fixationin crease the risk of these [2]. Reduction and osteosynthesis with intramedullary K-wire can be a good alternative toopen reduction and closed reduction and percutaneous pinning with low level of success due to especially excessive edema. The most important advantages of this technique are providing extra-articular closed reduction, eliminating the risk of devascularization of the radial head and nerve lesions seen with open reduction. With this technique, long-term immobilization is not required as in the closed reduction and early rehabilitation can be allowed.

## Conclusion

This method provides a short, safe and effective treatment without need for open reduction. Vessel-nerve injury in open reduction, difficulty in finding appropriate rotation axis of radial neck with ulna in internal fixation, especially rotation (supination, pronation) limitations in forearm due to synostosis andadhesions that can occur with open reduction can be overcome with this method. In our case, we have applied CIMP technique, which yielded good clinical results in pediatric radial neck fractures. We achieved excellent result. We believe that CIMP along with other surgical options must be found in mind for adult patients with radial neck fractures.
